# Vascular Effects of Ultrafine Particles in Persons with Type 2 Diabetes

**DOI:** 10.1289/ehp.1002237

**Published:** 2010-09-07

**Authors:** Judith C. Stewart, David C. Chalupa, Robert B. Devlin, Lauren M. Frasier, Li-Shan Huang, Erika L. Little, Steven M. Lee, Richard P. Phipps, Anthony P. Pietropaoli, Mark B. Taubman, Mark J. Utell, Mark W. Frampton

**Affiliations:** 1 Department of Medicine, University of Rochester Medical Center, Rochester, New York, USA;; 2 National Health and Environmental Effects Research Laboratory, U.S. Environmental Protection Agency, Research Triangle Park, North Carolina, USA;; 3 Department of Biostatistics and Computational Biology and; 4 Department of Environmental Medicine, University of Rochester Medical Center, Rochester, New York, USA

**Keywords:** air pollution, diabetes, platelets, ultrafine particles, vascular

## Abstract

**Background:**

Diabetes confers an increased risk for cardiovascular effects of airborne particles.

**Objective:**

We hypothesized that inhalation of elemental carbon ultrafine particles (UFP) would activate blood platelets and vascular endothelium in people with type 2 diabetes.

**Methods:**

In a randomized, double-blind, crossover trial, 19 subjects with type 2 diabetes inhaled filtered air or 50 μg/m^3^ elemental carbon UFP (count median diameter, 32 nm) by mouthpiece for 2 hr at rest. We repeatedly measured markers of vascular activation, coagulation, and systemic inflammation before and after exposure.

**Results:**

Compared with air, particle exposure increased platelet expression of CD40 ligand (CD40L) and the number of platelet-leukocyte conjugates 3.5 hr after exposure. Soluble CD40L decreased with UFP exposure. Plasma von Willebrand factor increased immediately after exposure. There were no effects of particles on plasma tissue factor, coagulation factors VII or IX, or D-dimer.

**Conclusions:**

Inhalation of elemental carbon UFP for 2-hr transiently activated platelets, and possibly the vascular endothelium, in people with type 2 diabetes.

Exposure to fine particulate air pollution (PM) is associated with increased cardiovascular mortality and increased hospitalizations for myocardial infarction and congestive heart failure (Frampton and Utell 2000; Peters et al. 2000, 2004; Pope et al. 2004; Utell et al. 2002). Exposure to PM has been linked with increased risk for venous thrombosis (Pope 2009). Diabetes increases susceptibility to cardiovascular disease and further increases the cardiovascular risks associated with ambient PM exposure (Goldberg et al. 2006; Liao et al. 2005; Zanobetti and Schwartz 2002). Among 92 subjects with diabetes in the Boston area, O’Neill et al. (2005) found that exposure to ambient PM was associated with reduced vascular reactivity and with elevated blood markers of vascular inflammation and injury (O’Neill et al. 2007). Black carbon exposure showed the strongest associations, which suggests that carbon-containing PM from traffic or power plants were most important in causing vascular effects. Exposure to another marker of traffic (nitrogen dioxide) has also been related to incident diabetes among women (Brook et al. 2008).

Carbonaceous ultrafine particles (UFP; < 100 nm) emitted from diesel-powered motor vehicles and other combustion sources may contribute to the cardiovascular effects associated with PM exposure (Frampton and Utell 2000). UFP deposit efficiently in the alveolar compartment of the lung (Chalupa et al. 2004; Daigle et al. 2003) and enter epithelial cells and the pulmonary vasculature (Geiser et al. 2005) where they may perturb vascular function (Frampton et al. 2006; Shah et al. 2008), activate platelets (Radomski et al. 2005), and promote thrombus formation (Nemmar et al. 2006). Panel studies of patients with coronary artery disease have shown relationships between UFP exposure and blood markers of platelet activation (Delfino et al. 2008; Rückerl et al. 2007). In healthy volunteers, inhalation of diesel exhaust containing 350 μg/m^3^ PM increased blood thrombus formation in an *ex vivo* perfusion chamber and also increased formation of platelet-neutrophil and platelet-monocyte aggregates, which are markers of platelet activation (Lucking et al. 2008). However, these exposures contained both particulate and gaseous emissions at relatively high concentrations, so the causative agent or agents is unknown.

We have shown previously that inhalation of UFP consisting of elemental carbon, as surrogates for UFP of combustion origin, subtly altered both pulmonary and systemic vascular function in healthy subjects (Frampton et al. 2006, 2007; Shah et al. 2008).

We hypothesized that inhalation of carbon UFP, without the gases and reactive organic molecules that are present in diesel exhaust, would activate vascular endothelium and blood platelets and promote coagulation in subjects with type 2 diabetes.

## Materials and Methods

The Research Subjects Review Board of the University of Rochester approved the study. Informed, written consent was obtained from all subjects.

### Subjects

Volunteers were 19 never-smokers 30–60 years of age with type 2 diabetes as defined by the World Health Organization (Alberti and Zimmet 1998). Subjects were recruited in Rochester, New York, using media advertisements; 38 persons were screened, and 19 of those screened met the study criteria and completed the study. We attempted to balance subject recruitment by sex and age (30–45 vs. 46–60 years old). Subjects were required to be on a stable medication regimen for at least 3 months prior to entry and were continued on the same regimen during the study. Exclusion criteria included clinical cardiovascular disease, major organ dysfunction, uncontrolled hypertension, frequent hypoglycemia, statin-type lipid-lowering medications (because of the anti-inflammatory effects of these medications), platelet-active drugs including aspirin, and occupational exposure to particles (e.g., welding, foundry work). Subjects were asked to avoid nonsteroidal antiinflammatory drugs and phosphodiesterase enzyme inhibitors during the study and were questioned regarding compliance at each visit.

### Protocol

This study was a double-blind, randomized, crossover design in which each subject inhaled both filtered air (0–10 particles/cm^3^) and elemental carbon UFP (50 μg/m^3^, count median diameter of 32 nm) by mouthpiece for 2 hr at rest, with at least 3 weeks separating the exposures. Subjects were admitted to the University of Rochester General Clinical Research Center the evening prior to exposure and remained overnight. Baseline blood measurements were performed at approximately 0900 hours the following morning. The exposures began at approximately 0930 hours. The first measurements occurred at 1200 hours (0.5 hr after completion of the exposure) and again at 1530 hours (3.5 hr after exposure). The subjects were then allowed to leave the center and returned the following 2 days for blood draws at 0900 hours (21 and 45 hr after the end of the exposure).

Details of particle generation and characterization have been published previously (Chalupa et al. 2002). The particles were generated in argon using an electric spark discharge between graphite electrodes in a commercial generator (Palas Aerosol Generator, model GFG-1000; Palas Co., Karlsruhe, Germany) modified to prevent off-gassing of organic materials from within the generator (McDonald et al. 2001). This produced particles consisting of > 95% elemental carbon, without metals. Particle number (condensation particle counters, Model 3220a; TSI, Inc., St. Paul, MN), mass (tapered element oscillating microbalance; Rupprecht and Patachnick, Albany, NY), and size distributions (Scanning Mobility Particle Sizer, Model 3071; TSI, Inc.) were monitored on both inspiratory and expiratory sides of the subject. The subject inhaled from a mouthpiece and wore a nose clip.

Blood was drawn from an antecubital vein with minimal trauma, with the subject resting and in a supine position. Lipid profiles, hemoglobin A1c (HgbA1c), microalbuminuria, and glucose were determined only at screening. Complete blood leukocyte and platelet counts were done at screening and all time points listed above. All were measured in the Strong Memorial Hospital Clinical Laboratories in Rochester, NY, using standard automated laboratory methodology.

### Immunocytometry

Immunofluorescence measurements were performed within 1 hr of phlebotomy. Antibodies are listed in Supplemental Material, Table 1 (doi:10.1289/ehp.1002237). Platelets were analyzed using a method adapted from Li et al. (1999) [see Supplemental Material, “Methods” (doi:10.1289/ehp.1002237)]. Circulating microparticles (MP) are cell fragments < 1 μm released from cells in response to cell injury, activation, or apoptosis (Simak and Gelderman 2006). They may increase in conditions involving vascular injury or inflammation. MPs were identified using size gating and surface markers of the cells of origin [see Supplemental Material, “Methods” (doi:10.1289/ehp.1002237)].

Platelet-leukocyte conjugates were characterized by gating the cellular region for simultaneous expression of leukocyte common antigen (CD45) and P-selectin (CD62P), an antigen expressed on activated platelets. Monocyte expression of surface markers was measured on a separately stained sample using light scatter and CD45 positivity, as described previously (Frampton et al. 2006). Flow analysis used CellQuest (BD Biosciences, San Jose, CA) and FloJo (TreeStar, Ashland, OR) software.

### Plasma markers

Plasma was stored at −80°C for subsequent analyses of soluble markers, as previously described (Beckett et al. 2005). We used commercial enzyme-linked immunoassays (ELISA) for most [see Supplemental Material, Table 2 (doi:10.1289/ehp.1002237)] and customized ELISAs to measure plasma tissue factor protein (sCD142) (Khorana et al. 2008) and soluble CD40 ligand (CD40L) (Rückerl et al. 2007).

### Data handling and statistics

This study used a standard, two-period crossover design in which each subject received both particles and air. The order of presentation was randomized separately for each sex. A washout period of at least 3 weeks between the exposures was included to minimize risk of carryover effects, and testing for these effects was therefore not included in the models.

Our approach was based on the method described by Jones and Kenward (2003), using mixed models for the analysis of crossover trials, with repeated measurements within treatment (exposure) periods. There are two types of covariance patterns among measurements from the same subject: correlations among measurements in the same treatment period at different time points, and among measurements from different treatment periods. We assumed that the between- and within-period treatment covariance structures were separable. To accommodate between-period treatment dependencies, we introduced subject effect in the mixed model. For the within-period treatment dependencies, we assumed an unstructured correlation structure, an approach commonly used for repeated measurements. In this analysis, we used SAS, version 9, PROC MIXED (SAS Institute Inc., Cary, NC).

We ran two models: Model 1 examined the primary hypothesis of treatment (exposure) effects and contained terms for treatment, period, time, and time-by-treatment interactions; model 2 explored whether the exposure effects were different between men and women and between two age groups: 30–45 years old and 45–60 years old. Model 2 contained terms for treatment, sex, age group, treatment-by-sex interactions, and treatment-by-age interactions. To retain a parsimonious model given the sample size, *n* = 19, we did not include time in this model. Model 2 was considered exploratory, because the study was not adequately powered to test these interaction effects. Both models included adjustment for the corresponding baseline measurement prior to exposure in each period. The time variable in model 1 is a categorical variable, which models the overall response trend over time within each period, and the time-by-treatment interactions examine the treatment effects over time. We examined *t-*tests from the mixed-models output and assessed significance of contrasts of treatment effects at different time points. We used linear regression analysis to determine relationships between outcomes and subject characteristics. Significance was achieved by *p* < 0.05. Because multiple comparisons were involved and some end points were related, the congruence and plausibility of the results were considered in interpreting significance. Marginally statistically significant differences that were isolated, implausible, or inconsistent with other findings were not considered meaningful.

## Results

Characteristics of the 19 subjects are found in Table 1. Sixty-nine percent (13/19) of the subjects were obese, with a body mass index (BMI) > 30 kg/m^2^. Triglycerides and the total cholesterol and high-density lipoprotein ratios were significantly higher among the men. Diabetic control was variable, with the HgbA1c ranging from 5.6 to 11.0%, but only one subject was within the desirable range of < 6.0%. Two subjects had significant microalbuminuria, only one of which was clinically significant, indicating early renal vascular injury. These subjects showed no marked differences from the group mean UFP responses and were included in the final analysis. All subjects had normal renal function, as determined by serum creatinine level. Two subjects were on no medications. As treatment for their diabetes, 12 subjects took oral agents, 2 took insulin, and 2 were on insulin and an oral agent. Three subjects were on angiotensin-converting enzyme inhibitors and three were on β-receptor blockers.

The mean (± SD) UFP exposure parameters were as follows: target mass concentration 50 μg/m^3^, measured mass concentration 50.7 ± 2.8 μg/m^3^, particle number concentration 10.0 ± 0.77 × 10^6^ particles/cm^3^, particle count median diameter 31.6 ± 1.5 nm, geometric SD 1.65 ± 0.01.

Subject queries revealed no exposure-related symptoms and an inability to discriminate between the exposures. There were no significant differences between exposures for the total white blood cell counts, leukocyte differential counts, or platelet counts (data not shown).

### Model 1 analysis: effect of UFP exposure

**Figure 1:**
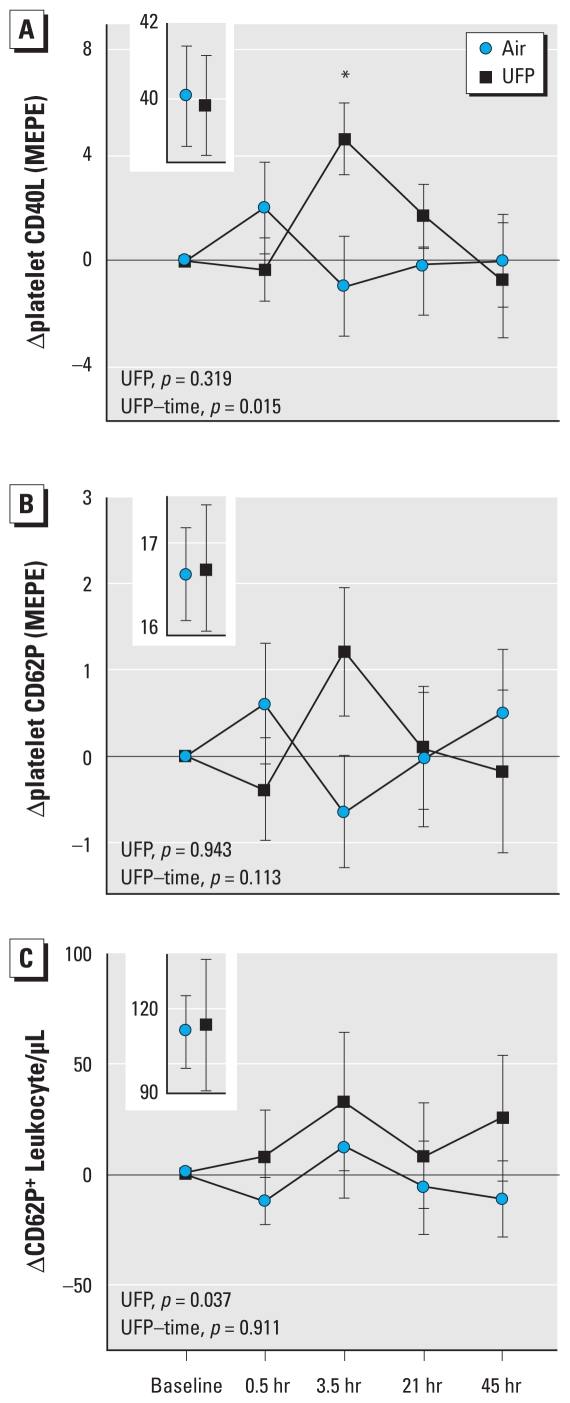
Markers expressed on the platelet surface with activation indicate differences in response to UFP relative to air. (*A*) Platelet expression of CD40L (*n* = 16) shows a significant UFP–time interaction, with the greatest effect 3.5 hr after exposure (*t-test comparing UFP and air at 3.5 hr, correcting for baseline, *p* = 0.004). (*B*) Expression of CD62P (*n* = 19) shows a similar but nonsignificant increase at 3.5 hr. (*C*) The number of leukocytes expressing CD62P (*n* = 18), which is indicative of conjugation with activated platelets and/or platelet MP, shows a significant difference in response after UFP versus air exposure. Preexposure values are shown in the insets. Expression is measured in molecules of equivalent phycoerythrin (MEPE).

Platelet activation was assessed by measuring changes in platelet surface expression of CD40L and CD62P and changes in the number of platelet-leukocyte conjugates relative to the baseline values of each exposure. Mean CD40L expression increased significantly 3.5 hr after exposure to UFP compared with air (Figure 1A), with a significant UFP–time interaction, indicating the UFP effect on CD40L expression differed over time. CD40L expression 3.5 hr after UFP was increased from baseline levels in 9 of the 13 subjects with complete data [see Supplemental Material, Figure 1 (doi:10.1289/ehp.1002237)]. The increase in platelet expression of CD62P (Figure 1B) was not statistically significant (UFP–time interaction, *p* = 0.11), although the increases in CD62P 3.5 hr after UFP exposure (*p* = 0.09) correlated strongly by paired regression with those of CD40L (*r* = 0.94, *p* < 0.001). UFP exposure also significantly increased the number of leukocytes expressing CD62P, which indicates conjugation of leukocytes with activated platelets and/or platelet MP (Figure 1C). Detailed results for these surface markers are provided in Supplemental Material, Table 3 (doi:10.1289/ehp.1002237).

Tissue factor, a measure of endothelial perturbation, was measured as expression on monocytes and platelets, as numbers of tissue factor expressing MP, and as plasma protein [see Supplemental Material, Table 4 (doi:10.1289/ehp.1002237)]. Although none showed statistically significant differences between UFP and air exposure, all increased, except the plasma levels, 3.5 hr after UFP exposure [see Supplemental Material, Figure 2 (doi:10.1289/ehp.1002237)]. The plasma tissue factor increased immediately after UFP exposure and then decreased. The increase in platelet tissue factor expression 3.5 hr after UFP exposure (relative to baseline) approached significance [see Supplemental Material, Figure 2A (doi:10.1289/ehp.1002237)] (*p* = 0.07) and correlated strongly with the previously mentioned significant increases, at this time point, in expression of platelet CD40L (*r* = 0.91, *p* < 0.001). We found no significant effects of UFP exposure on the numbers of platelet or endothelial MPs [see Supplemental Material, Table 5 (doi:10.1289/ehp.1002237)].

**Figure 2:**
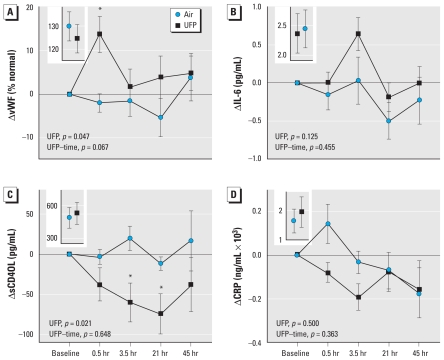
Changes in plasma soluble markers indicate different responses to UFP exposures than to air. (*A*) von Willebrand factor (*n* = 19) shows an overall significant UFP effect, with the greatest effect 0.5 hr after exposure (*t-test comparing UFP and air at 0.5 hr correcting for baselune, *p* = 0.003). (*B*) IL-6 (*n* = 19) increased nonsignificantly after UFP exposure. (*C*) Soluble CD40L (*n* = 19) shows a significant overall decrease after UFP versus air exposure and significant decreases at 3.5 and 21 hr after UFP exposure corrected for baseline (*t-test *p* = 0.029 and 0.022, respectively). (*D*) CRP (*n* = 19) decreased nonsignificantly after UFP exposure. Preexposure values are shown in the insets.

We measured plasma concentrations of von Willebrand factor (vWF) (Figure 2A) as a marker of endothelial activation or injury. Mean vWF increased significantly 0.5 hr after UFP exposure compared with baseline levels (Figure 2A)—15 of 18 subjects with complete data showed the increase 0.5 hr after UFP [see Supplemental Material, Figure 3 (doi:10.1289/ehp.1002237)]. Plasma interleukin-6 (IL-6) (Figure 2B) increased relative to baseline 3.5 hr after UFP exposure relative to baseline (*t*-test *p* = 0.09) but was not statistically significant (model 1 UFP effect, *p* = 0.13). Soluble CD40L decreased significantly after UFP exposure (Figure 2C) (UFP effect, *p* = 0.021), a direction that was opposite of what we expected. There were no significant UFP effects on soluble C-reactive protein (CRP) (Figure 2D), E-selectin, L-selectin, CD62P, intercellular cell adhesion molecule (ICAM-1), vascular cell adhesion molecule (VCAM-1), serum amyloid A (SAA), or the coagulation markers (D-dimer, Factors VII and IX) [see Supplemental Material, Table 6 (doi:10.1289/ehp.1002237)].

### Model 2 analysis: interactions of UFP exposure with sex and age

**Figure 3:**
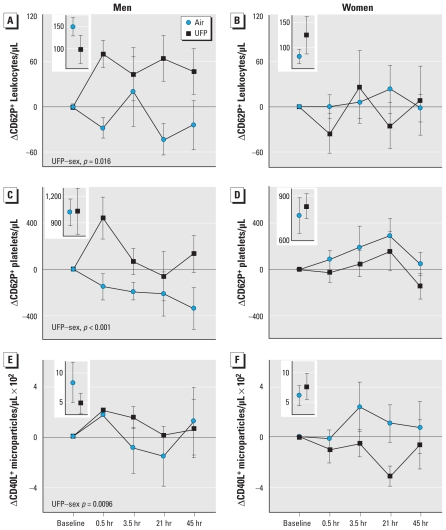
Sex-related responses. (*A–B*) Changes in the number of leukocytes expressing CD62P in men (*A*, *n* = 8) and women (*B*, *n* = 10). Model 2 analysis shows a significant overall UFP effect (*p* = 0.034) and a significant UFP–sex interaction. (*C–D*) Changes in the number of platelets expressing CD62P in men (*C*, *n* = 9) and women (*D*, *n* = 10). Model 2 analysis shows a significant UFP–sex interaction. (*E–F*) Changes in the number of MP expressing CD40L in men (*E*, *n* = 6) and women (*F*, *n* = 9). Model 2 analysis shows a significant UFP–sex interaction, with increases in men and decreases in women after UFP exposure. Preexposure values are shown in the insets.

**Figure 4:**
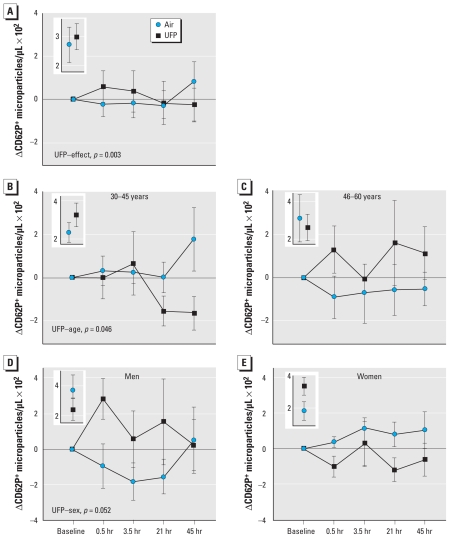
Age-related responses. Change in the number of MP expressing CD62P for all subjects (*A*); subjects 30–45 years old (*B*); subjects 46–60 years old (*C*); male subjects (*D*); and female subjects (*E*). Model 2 analysis showed a significant overall UFP treatment effect and a significant UFP–age interaction. There was a marginally significant UFP–sex interaction. Preexposure values are shown in the insets.

A secondary objective of this study was to determine if subject sex and age influenced the effects of UFP. Three outcomes demonstrated significant UFP exposure–sex interactions (Figure 3): the numbers of leukocytes and platelets expressing CD62P and those of MP expressing CD40L. For all three outcomes, men increased after UFP exposure relative to women. Only one variable showed a significant UFP–age interaction. The number of CD62P^+^ MP increased in subjects who were 45–65 years old but decreased in those who were 30–45 years old after UFP exposure (Figure 4B–C). This variable also showed an overall UFP exposure effect with the model 2 analysis (Figure 4A), and a marginally significant exposure–sex interaction (Figure 4D,E). Other variables showed no significant sex or age interactions, and overall, there did not appear to be convincing evidence for sex or age differences in UFP effects on platelet activation (data not shown).

We found no significant relationships between any of the significant UFP exposure effects and BMI, total cholesterol, high- or low-density lipoprotein, HgbA1c, or microalbuminuria.

## Discussion

To our knowledge, this is the first clinical study of people with type 2 diabetes inhaling UFP. We found that inhalation of freshly generated elemental carbon UFP, when compared with filtered air, initiated several changes. Plasma vWF transiently increased 0.5 hr after exposure. Platelet expression of CD40L and the number of platelet-leukocyte conjugates increased 3.5 hr after exposure. On the other hand, soluble plasma CD40L decreased after exposure. There were significant sex interactions in the UFP effects on platelet-leukocyte conjugates, CD62P^+^ platelets, and CD40L^+^ MP, but not for other end points. UFP exposure increased the number of activated platelet MP in subjects 45–65 years old but decreased them in subjects 30–45 years old, the only significant age-related effect. These findings are consistent with effects of inhaled UFP on platelet activation.

The markers examined in this study reflect risk for atherosclerotic vascular disease. For example, increased plasma levels of vWF, which can be released by both vascular endothelial cells and platelets, have been linked to endothelial dysfunction and increased cardiovascular disease risk (Lee et al. 2005; Roldán et al. 2005; Zareba et al. 2001). Both vWF and IL-6 are released into the plasma during acute coronary syndromes (Montalescot et al. 1998) and are predictive of adverse outcomes (Lee et al. 2005). CD40L–CD40 interactions appear important in the pathogenesis of atherosclerosis (Phipps 2000). Surface expression of CD40L is considered a marker of platelet activation (Henn et al. 1998) and has been considered a marker of atherosclerotic vascular disease (Garlichs et al. 2001; Stumpf et al. 2003). Tissue factor appears to have an important role in mediating the increased thrombogenicity in persons with type 2 diabetes (Sambola et al. 2003). Circulating MP that are thrombogenic often express tissue factor, and may enhance activation of platelets, leukocytes, and endothelial cells (Jimenez et al. 2003; Morel et al. 2006). Patients with acute coronary syndromes show increased levels of circulating platelet-monocyte aggregates (Freedman and Loscalzo 2002). Thus, the observed increases in vWF, platelet expression of CD40L, and platelet-leukocyte conjugates are consistent with platelet activation in response to UFP exposure.

An increase in platelet expression of CD40L was detected 3.5 hr after UFP exposure, yet soluble CD40L levels decreased, reaching a nadir 21 hr after exposure. The reason for this inverse relationship is unknown. Some studies have associated increased soluble CD40L levels with cardiovascular disease (Garlichs et al. 2001; Schönbeck et al. 2001), whereas others have not found such a relationship (Lim et al. 2004; Stumpf et al. 2003). This may reflect the complex relationships between platelet and plasma soluble CD40L that were reported by Mason et al. (2005). They found negative correlations between platelet surface and plasma soluble CD40L in patients with cardiovascular disease. Platelet CD40L, but not soluble CD40L, proved to be a reliable marker of platelet activation. In our study, it is possible that UFP exposure caused both an increase in platelet expression of CD40L and a more rapid clearance of CD40L from the plasma. It is also possible that platelet activation does not always lead to release of CD40L into the plasma.

We compared the magnitude of the changes in platelet CD40L and plasma vWF observed in our study with previously published measurements in patients to understand the potential risk of UFP effects in susceptible people. For platelet expression of CD40L, we observed a mean 13% increase [44 vs. 40 MEPE (molecules of equivalent phycoerythrin) units] 3.5 hr after UFP exposure. Garlichs et al. (2001) found that platelet CD40L expression was 300% higher (11 vs. 3.6 fluorescence intensity units, *p* < 0.001) in patients with unstable angina relative to patients with stable angina. Stumpf et al. (2003) found a 266% increase (35.6 vs. 13.4 fluorescence intensity units, *p* < 0.001) in platelet CD40L expression in patients with ischemic cardiomyopathy compared with patients with coronary artery disease without cardiomyopathy. For vWF, we observed a mean increase of 10% (138 vs. 125 percentage units) 0.5 hr after UFP exposure. Horii et al. (2008) found that vWF levels were 40% higher (211 vs. 151 percentage units, *p* < 0.05) in elderly patients with acute myocardial infarction than in age-matched healthy volunteers. Thus, the mean changes in CD40L expression and vWF in our study were smaller than, but within an order of magnitude of, the increases seen in patients with clinical cardiovascular disease.

Our findings add to previous observations in panel and clinical studies. In panel studies, exposure to ambient air pollution was associated with increased blood levels of vWF in patients with diabetes (O’Neill et al. 2007). However, in patients with coronary artery disease, plasma CD40L was found to be increased, rather than decreased, in association with air pollution exposure (Rückerl et al. 2007). Panel studies have also shown pollutant-related increases in IL-6 and CRP (Delfino et al. 2008; Dubowsky et al. 2006) and reductions in platelet counts (Rückerl et al. 2007) and vascular endothelial function (O’Neill et al. 2005; Schneider et al. 2008). In clinical studies, inhalation of diesel engine exhaust caused vascular dysfunction (Mills et al. 2005; Peretz et al. 2008), increased platelet-leukocyte aggregation (Lucking et al. 2008), and increased cardiac ischemia during exercise in men with stable coronary artery disease (Mills et al. 2007). However, in another diesel inhalation study, Carlsten et al. (2007) did not find thrombogenic effects of diesel exhaust in healthy people.

Inhalation of concentrated ambient fine PM did not alter endothelial function or blood fibrinolytic function in patients with coronary artery disease (Mills et al. 2008), possibly because the PM concentrations were lower (190 μg/m^3^) than in the diesel studies and of largely nontraffic origin. Inhalation of concentrated ambient UFP increased plasma levels of D-dimer, a fibrin degradation product, indicating increased formation and degradation of fibrin clot (Samet et al. 2009). However, there were no effects on a variety of additional markers of coagulation and fibrinolysis that were measured in that study, and D-dimer levels were unaffected in our study. In previous studies of healthy subjects in our laboratory (Pietropaoli et al. 2004), inhalation of elemental carbon UFP for 2 hr did not alter platelet counts or plasma levels of IL-6, vWF, fibrinogen, or factor VII. Inconsistent findings across these clinical studies may be related, in part, to differing subject populations, exposures, and measurement techniques.

Our current study extends previous studies in demonstrating that inhalation of elemental carbon UFP, at mass concentrations much lower than the diesel exhaust studies, induces platelet and possibly leukocyte and endothelial activation in people with type 2 diabetes. The exposure mass concentration used in our study, 50 μg/m^3^, is relevant to concentrations encountered on or near roadways in urban environments, where the fine particle mass can exceed 100 μg/m^3^ (HEI Panel on the Health Effects of Traffic-Related Air Pollution 2010). The laboratory-generated particles used in our study did not contain metals or organic carbon, both of which have been implicated as components that drive PM health effects. Thus, additional mechanisms must be considered to explain the vascular effects of UFP, such as surface chemistry or direct translocation of particles to the vascular space.

Our study has limitations. The UFP generated in this study represent an imperfect surrogate of ambient air UFP, and the observed effects therefore may not be representative of those occurring with exposures to ambient air pollution. Because ambient UFP contain organic carbon and other chemical species that may mediate toxicity, it is possible that the vascular effects of ambient UFP may be greater than for the elemental carbon UFP used in this study. The exposure concentrations were considerably higher than ambient UFP concentrations, but were relevant to specific environments, such as on or near busy roads (Kittleson et al. 2004). Although subjects were hospitalized overnight prior to each experimental exposure, it remains possible that uncontrolled environmental exposures, before and after the experimental exposures, affected their responses. However, influences of ambient exposures would likely have biased the results toward the null. We studied a relatively small number of persons with stable type 2 diabetes in a specific age range, without clinical cardiovascular disease, who were not on lipid-lowering statin drugs; our findings may not be representative of individuals with type 2 diabetes in general, many of whom take statin-type drugs. Furthermore, the study design does not allow us to determine whether diabetes or other subject characteristics confer increased susceptibility to the observed effects.

In summary, in people with type 2 diabetes, inhalation of 50 μg/m^3^ elemental carbon UFP for 2 hr at rest caused transient activation of blood platelets, with possible associated activation of blood leukocytes and vascular endothelium. These effects, although transient and small in magnitude, suggest an acute vascular insult with prothrombotic consequences that could increase the risk for an acute cardiovascular event in people with overt atherosclerotic vascular disease. This may help explain the observed associations between PM exposure and acute cardiovascular effects in people with diabetes.

## Figures and Tables

**Table 1:** Subject characteristics measured at screening.^a^

			Age groups	
Parameter	Men	Women	30–45 years	46–60 years
No. of subjects (*n*)	9	10	10	9
Age (years)	48.3 ± 8.8	43.7 ± 10.0	38.0 ± 1.4	54.7 ± 1.3
Race/ethnicity (*n*)				
African American	0	3	2	1
Asian	1	0	0	1
Hispanic	0	1	0	1
White	8	6	8	6
BMI (kg/m^2^)	34.7 ± 5.2	31.1 ± 5.5	32.8 ± 2.1	32.8 ± 1.5
Cholesterol (mg/dL)	191.0 ± 36.7	168.5 ± 31.9	181.7 ± 11.9	176.3 ± 11.4
Triglycerides (mg/dL)	185.9 ± 55.1	102.9 ± 80.5*	142.6 ± 31.0	141.8 ± 20.1
High-density lipoprotein (mg/dL)	40.6 ± 7.0	47.5 ± 8.1	44.7 ± 2.5	43.7 ± 2.9
Low-density lipoprotein (mg/dL)	113.2 ± 38.0	100.5 ± 27.8	108.4 ± 12.1	104.4 ± 9.3
Cholesterol–high-density lipoprotein ratio	4.8 ± 0.8	3.7 ± 0.9*	4.2 ± 0.3	4.2 ± 0.4
HgbA1c (mmol/mol)	7.6 ± 1.1	7.7 ± 1.8	7.6 ± 0.6	7.7 ± 0.4
Glucose (mg/dL)	151.4 ± 34.8	168.4 ± 94.0	174.7 ± 28.9	144.4 ± 12.3
Microalbuminuria (mg/dL)	20.2 ± 58.4 (0.8 ± 0.5)^b^	2.9 ± 5.9 1.0 ± 1.1^c^	18.6 ± 17.5 1.1 ± 0.3^b^	2.8 ± 2.1 0.7 ± 0.2^c^

a Data are given as mean ± SD.

b Without one male outlier at 176.0 mg/dL.

c Without one female outlier at 19.3 mg/dL.

* *p* < 0.05 between sexes.
